# The relationship between addiction to mobile phone and sense of loneliness among students of medical sciences in Kermanshah, Iran

**DOI:** 10.1186/s13104-019-4728-8

**Published:** 2019-10-22

**Authors:** Hale Jafari, Abas Aghaei, Alireza khatony

**Affiliations:** 1 Tehran School of Nursing and Midwifery, Tehran University of Medical Sciences, Tehran, Iran; 2 Social Determinants of Health Research Center, Research Institute for Health Development, Kurdistan University of Medical Sciences, Sanandaj, Iran; 3 Clinical Research Development Center, Imam Reza Hospital, Kermanshah University of Medical Sciences, Kermanshah, Iran; 4 Health Institute, Social Development and Health Promotion Research Center, Kermanshah University of Medical Sciences, Kermanshah, Iran

**Keywords:** Addiction, Cell phones, Loneliness, Medical, Students

## Abstract

**Objective:**

Addiction to mobile phone is one of the negative consequences of excessive use of this device. This disorder may also be related to individuals’ loneliness and may reduce or increase the sense of loneliness. Therefore, the present study aimed to investigate the relationship between addiction to mobile phone and sense of loneliness among medical sciences students.

**Results:**

In this descriptive-analytical study, 439 students entered the study by stratified random sampling. The study tool was the mobile phone addiction and SELSA’s sense of loneliness questionnaires. The average score of mobile phone addiction in boys and girls was 73.77 ± 11.48 and 74.64 ± 12.28 from 100, respectively. There was no significant difference between them. According to the rating of mobile phone addiction, 17.8% of the students were in the range of moderate dependency and 10.9% of them were in the range of extreme dependency. Also, 71.3% of the students were identified as mobile phone addicts. The average score of sense of loneliness in boys and girls was 43.22 ± 5.16 and 42.82 ± 5.30, out of 105, respectively. There was no significant difference between them. There was a significant and negative correlation between the scores of mobile phone addiction and sense of loneliness.

## Introduction

The use of mobile phones has become extensively popular in recent years [1]. Despite the many benefits that mobile phones have as an opportunity, excessive use of them can cause various problems such as mobile addiction [2, 3]. Mobile phone addiction can be mentioned as one of the most important social problems caused by excessive use of mobile phones [4]. Griffiths describes technological addiction as a behavioral and non-chemical addiction that is created by interaction with device [5]. Mobile addiction has been introduced as a type of technology-related disorders in the 5th International Classification of Mental Disorders [4]. Reduced social relations and increased sense of loneliness are the likely outcomes of excessive mobile phone use [3]. Loneliness is considered as one of the strong predictors of mobile phone addiction [6]. Excessive use of mobile phone, by reducing the time allocated to social relations, can create a sense of loneliness [7]. Since face-to-face communication usually reduces the sense of loneliness in individuals [7, 8], the extensive use of mobile phone, by replacing real relationships with virtual and weak relationships, affects the sense of loneliness [9]. In this regard, Mansourian et al., have found a negative and significant relationship between the sense of loneliness and mobile phone addiction, and have introduced mobile phone use as a reducing factor for loneliness [3]. However, some other studies have found a positive and significant relationship between the sense of loneliness and the extent of mobile phone use in students [8–10]. Considering the importance of mobile phone addiction and the lack of studies on the relationship between addiction to mobile phone and sense of loneliness among the students of medical sciences in Kermanshah, this study was done.

## Main text

### Methods

#### Study design

This study had a descriptive-analytical design.

#### Study questions

1. What is the extent of mobile phone addiction among the students?
2. What is the degree of sense of loneliness among the students?
3. What is the relationship between mobile phone addiction and sense of loneliness among students?.

#### Sample and sampling method

The study population included all students of Kermanshah University of Medical Sciences (KUMS). The study sample consisted of 439 students who were studying at medical, dentistry, pharmacy, health, nursing and midwifery and allied medical sciences faculties. Sample size was calculated to be 399 students considering the sample size in the previous study [3] and also using the sample size formula for estimating a ratio. Furthermore, a confidence interval of 95% and a precision of 0.025 were also considered, and 10% sample drop was estimated. Therefore, in total 439 students entered the study. The inclusion criteria comprised of studying at one of the university’s affiliated faculties and giving written consent to participate in the study. Stratified random method was used for sampling and each faculty affiliated to the KUMS, formed a stratum. Inside each stratum, random sampling was done using a random number table. According to the number of students in each faculty, a percentage of them were selected as the sample of the entire university students. For this purpose, the list of students of each faculty was first obtained from the Education Department. Then, using a table of random number, 439 students were recruited.

#### Instruments

The tool used in this study was a three-part questionnaire. The first part was related to demographic information. The second and third parts of the questionnaire included the standard questionnaire for mobile phone addiction and the social and emotional loneliness scale for adults (SELSA), respectively. The personal information form included questions about age, sex, field of study, educational level, duration of mobile phone use by year, average number of daily calls and messages sent and received, and average monthly credit bought.

The mobile phone addiction questionnaire was designed by Young Koo, and its Cronbach’s alpha has been calculated to be 0.92 [11]. The Persian version of the questionnaire is psychometrically validated [12]. This questionnaire has 20 questions that are categorized and scored in three areas of withdrawal/tolerance (7 questions), life dysfunction (6 questions) and compulsion/persistence (7 questions). Accordingly, individuals are ranked in one of the “mobile phone addicts”, “severe user” and “average user” groups. The responses are in a five-option Likert’s scale, including “no, very little, little, very, and very much”, which are scored from one to five, respectively. The total score of each field is considered as the score of that field, and the total score of 20 questions is considered as the total score of the individual’s mobile phone addiction. The score range of the questionnaire is 20–100, with score of 70 or more indicating the addiction, between 63 and 70 indicating the high use, and less than 63 indicating the moderate use of mobile phone.

The social and emotional loneliness scale for adults was designed by Ditommaso et al. [13]. Subcategories of this questionnaire include social, family and romantic, all of which are divided into two emotional (romantic and family subcategories) and social dimensions. The validity and reliability of this tool have been verified by Jowkar and Salimi and its alpha coefficient in romantic, social and family dimensions has been 0.92, 0.84 and 0.78, respectively, which indicates the reliability of the questionnaire [14]. SELSA’s emotional and social loneliness scale for adults has 15 items with a seven-point response in Likert’s scale. In front of each item, numbers from one to seven are written with 1 indicating “strongly disagree” and 7 indicating “strongly agree”. Items 2, 5, 3, 6, 8, 14, 12, 11, 9 are scored in reverse order. The range of scores is between 15 and 105, and the higher score indicates more sense of loneliness.

#### Data gathering

After obtaining approval from the University’s Ethics Committee, the researcher attended the University’s affiliated faculties for sampling based on the student’s classroom program. At first, the study objectives were explained to the participants and consent was obtained from them. Then, the questionnaires were given to the participants.

#### Data analysis

Data were analyzed by the SPSS v.18.0 software using descriptive and inferential statistics. To assess data normalization, Kolmogorov–Smirnov test was used and the results showed that, except for the total score of sense of loneliness, other variables did not follow the normal distribution. The Spearman correlation coefficient test was used to determine the relationship between mobile phone addiction and sense of loneliness. The significance level was considered less than 0.05.

#### Ethical considerations

The Ethics Committee of the University approved the study. The emphasis was placed on the confidentiality of personal details and the responses of participants. A written informed consent was obtained from the participants.

### Results

The mean age of students was 23.18 ± 3.39 years and 54.2% (n = 238) of them were female. Most of the subjects were studying at the faculty of medicine (29.4%, n = 129), and they were mainly undergraduate students (57.4%, n = 252) as well as students of general medicine (n = 169, 38.5%). The average history of mobile phone use among the subjects was 6.19 ± 2.47 years. The average number of daily call was 5.59 ± 6.41 calls, and the average number of daily SMS was 14.79 ± 29.18 SMS. Also, the average call duration was 9.59 ± 10.04 min/day. The subjects were spending an average of $ 5.3 a month to buy mobile phone credit (Table 1).

**Table 1:** Demographic characteristics of study subjects

Variables	Mean ± SD
Age (year)	23.18 ± 3.39
Duration of mobile usage (year)	6.19 ± 2.47
Number of daily calls	5.59 ± 6.41
Daily call duration (min)	10.04 ± 9.59
Number of daily SMS	5.80 ± 5.30

Variables	Number (%)
Sex	
Male	201 (45.8)
Female	238 (54.2)
School	
Public health	90 (20.5)
Nursing and midwifery	61 (13.5)
Medicine	129 (29.4)
Allied medical sciences	114 (26)
Pharmacy	27 (6.2)
Dentistry	18 (4.1)

*SMS* short message system, *SD* standard deviation

The average score of mobile phone addiction in boys and girls was 73.77 ± 11.48 and 74.64 ± 12.28 from 100, respectively. There was no significant difference between them. Based on mobile phone addiction scale, 17.8% (n = 78) students had moderate dependency, 10.9% (n = 48) had sever dependency, and 71.3% (n = 313) were identified as mobile addicts. The average score of students in the withdrawal/tolerance domain was 25.9 ± 4.89, in the life dysfunction domain was 21.68 ± 4.32 and in the compulsion/persistence domain was 26.63 ± 3.94.

The average score of sense of loneliness in boys and girls was 43.22 ± 5.16 and 42.82 ± 5.30, out of 105, respectively. There was no significant difference between them. This score was 15.57 ± 3.28 for social dimension, 14.44 ± 2.99 for the family dimension, and 14.46 ± 2.88 for the romantic dimension. The results of examining the correlation between mobile phone addiction and its constituents with loneliness and its constituents showed that, except for two cases (the domain of coercion and persistence in mobile phone addiction with family and romantic dimensions of loneliness), other domains had a negative and significant correlation with each other. This negative and significant correlation was also observed in the total score of mobile phone addiction and total loneliness score (*p *< 0.001) (Table 2).

**Table 2:** Relationship between dimensions of mobile phone addiction and loneliness

Dimensions of cell phone addiction	Dimensions of loneliness						Loneliness (total)	
	Social		Family		Romantic			
	*p*	r^a^	*p*	r	*p*	r	*p*	r
Withdrawal/tolerance	< 0.001	− 0.282	< 0.001	− 0.206	0.001	− 0.153	< 0.001	− 0.332
Life dysfunction	< 0.001	− 0.304	< 0.001	− 0.226	< 0.001	− 0.190	< 0.001	− 0.360
Compulsion/persistence	< 0.001	− 0.151	0.273	− 0.052	0.551	− 0.029	0.005	− 0.133
Cell phone addiction (total)	< 0.001	− 0.282	< 0.001	− 0.196	0.001	− 0.152	< 0.001	− 0.324

^a^Based on Spearman correlation coefficient test

Age and daily call duration were directly and positively correlated with Cell phone addiction and its dimensions, and these correlations were significant (p < 0.05). The number of daily SMS was directly and positively correlated with the score of Cell phone addiction score and dimension of Withdrawal/tolerance. Also, there was a direct and positive correlation between daily contact with social dimension, and number of daily SMS with family dimension of Loneliness, and these correlations were significant (p < 0.05) (Table 3).

**Table 3:** Correlation between age and some variables related to mobile use with cell phone addiction and loneliness as well as their dimensions

	Duration of mobile usage (year)		Number of daily calls		Daily call duration (min)		Number of daily SMS		Age	
	*p*	r^a^	*p*	r	*p*	r	*p*	r	*p*	r
Withdrawal/tolerance	0.181	0.064	0.351	0.044	< 0.001	0.211	0.025	0.107	0.033	0.102
Life dysfunction	0.296	0.050	0.458	0.035	< 0.001	0.213	0.052	0.093	0.005	0.135
Compulsion/persistence	0.206	0.060	0.773	− 0.014	< 0.001	0.227	0.060	0.090	0.015	0.116
Cell phone addiction (total)	0.193	0.062	0.493	0.033	< 0.001	0.238	0.027	0.105	0.012	0.120
Dimensions of loneliness										
Social	0.757	0.015	0.787	− 0.013	0.047	0.097	0.832	0.010	0.159	0.067
Family	0.121	0.074	0.404	0.040	0.124	0.073	0.046	0.095	0.618	0.024
Romantic	0.862	0.008	0.302	0.049	0.052	− 0.093	0.927	− 0.004	0.799	− 0.012
Loneliness (total)	0.151	0.068	0.264	0.053	0.208	0.060	0.111	0.076	0.275	0.052

*SMS* short message system

^a^Based on Spearman correlation coefficient test

### Discussion

The aim of this study was to investigate the relationship between addiction to mobile phone and sense of loneliness among students of medical sciences in Kermanshah, Iran. The results showed that most of the students (71.3%) had mobile phone addiction. The rate of mobile phone addiction has been reported quite differently among students in different countries. For example, this figure in China was 13.5% [6], in Iran was 1.2% [15], and in Korea was 0.9% [16]. Since the sample size and tools used to measure mobile phone addiction have been appropriately chosen in all of the above studies, the difference in the rate of addiction to mobile phone can be related to the differences in the study population or the time of study. We found that the degree of loneliness that students felt was at average level. In some studies, students’ sense of loneliness has been reported at moderate level [3, 6], which is consistent with our results. We found a significant and negative relationship between mobile phone addiction and the sense of loneliness, which meant that people who had more addiction to their mobile phone had less sense of loneliness. Mansourian et al. have also found a negative and significant correlation between mobile phone addiction and sense of loneliness [3], but in most studies, a positive and significant relationship has been found between mobile phone addiction and sense of loneliness [6, 9, 10, 17]. Bhardwaj quoted Jin and Park and stated that, extensive phone calls are associated with the higher sense of loneliness [7]. Bian and Leung believed that, individuals are not only interested in face-to-face communication with others, but also prefer to communicate with others through messages and social networks [6]. In our view, suggesting ways to reduce the addiction of students to mobile phone, replacing virtual communication with realistic relationships, and providing programs to reduce or eliminate individuals’ sense of loneliness are essential, rather than using mobile phones.

Our results are worth examining from this dimension as it seems that in the past, individuals with limited social relationships were more likely to use mobile phone to avoid face-to-face communication, but according to our results, students who were more addicted to their mobile phone felt less lonely. This finding suggests that, the mobile phone has succeeded in removing students’ loneliness and replacing face-to-face family, emotional, and social relationships. Therefore, further studies are suggested on this subject and other subjects such as; changes made in the definition of loneliness, and review of loneliness measuring tools.

Our results showed that most students of medical sciences were addicted to their mobile phone, and this way, they were experiencing less sense of loneliness. The redefinition of the concept of sense of loneliness and its dimensions in the present time, the design of new tools for measuring the sense of loneliness, and conducting qualitative studies to explain students’ understanding of loneliness are suggested.

## Limitations

Completion of the questionnaires by self-reporting was one of the limitations that could affect the accuracy of our results. Regarding the type of our study, it is difficult to explain the cause and effect relationships between the main variables of the study.

## Data Availability

Data is available by contacting the corresponding author.

## Abbreviation

KUMS: Kermanshah University of Medical Sciences
